# Verteporfin-Loaded Anisotropic Poly(Beta-Amino Ester)-Based Micelles Demonstrate Brain Cancer-Selective Cytotoxicity and Enhanced Pharmacokinetics

**DOI:** 10.2147/IJN.S231167

**Published:** 2019-12-23

**Authors:** James G Shamul, Sagar R Shah, Jayoung Kim, Paula Schiapparelli, Carla A Vazquez-Ramos, Ben J Lee, Kisha K Patel, Alyssa Shin, Alfredo Quinones-Hinojosa, Jordan J Green

**Affiliations:** 1 Department of Biomedical Engineering, Johns Hopkins School of Medicine, Baltimore, MD 21231, USA; 2 Translational Tissue Engineering Center and Institute for NanoBioTechnology, Johns Hopkins School of Medicine, Baltimore, MD 21231, USA; 3 Department of Neurosurgery, Mayo Clinic, Jacksonville, FL 32224, USA; 4 Department of Neurosurgery, Johns Hopkins Hospital, Baltimore, MD 21231, USA; 5 Department of Oncology, The Sidney Kimmel Comprehensive Cancer, and The Bloomberg~Kimmel Institute for Cancer Immunotherapy, Johns Hopkins School of Medicine, Baltimore, MD 21231, USA; 6 Department of Ophthalmology, Department of Materials Science and Engineering, and Department of Chemical and Biomolecular Engineering, Johns Hopkins University, Baltimore, MD 21231, USA

**Keywords:** GBM, verteporfin, micelle, anisotropic, poly(ethylene glycol), PEG, poly(beta-amino ester), PBAE

## Abstract

**Background:**

Nanomedicine can improve traditional therapies by enhancing the controlled release of drugs at targeted tissues in the body. However, there still exists disease- and therapy-specific barriers that limit the efficacy of such treatments. A major challenge in developing effective therapies for one of the most aggressive brain tumors, glioblastoma (GBM), is affecting brain cancer cells while avoiding damage to the surrounding healthy brain parenchyma. Here, we developed poly(ethylene glycol) (PEG)-poly(beta-amino ester) (PBAE) (PEG-PBAE)-based micelles encapsulating verteporfin (VP) to increase tumor-specific targeting.

**Methods:**

Biodegradable, pH-sensitive micelles of different shapes were synthesized via nanoprecipitation using two different triblock PEG-PBAE-PEG copolymers varying in their relative hydrophobicity. The anti-tumor efficacy of verteporfin loaded in these anisotropic and spherical micelles was evaluated in vitro using patient-derived primary GBM cells.

**Results:**

For anisotropic micelles, uptake efficiency was ~100% in GBM cells (GBM1A and JHGBM612) while only 46% in normal human astrocytes (NHA) at 15.6 nM VP (p ≤ 0.0001). Cell killing of GBM1A and JHGBM612 vs NHA was 52% and 77% vs 29%, respectively, at 24 hrs post-treatment of 125 nM VP-encapsulated in anisotropic micelles (p ≤ 0.0001), demonstrating the tumor cell-specific selectivity of VP. Moreover, anisotropic micelles showed an approximately fivefold longer half-life in blood circulation than the analogous spherical micelles in a GBM xenograft model in mice. In this model, micelle accumulation to tumors was significantly greater for anisotropic micelle-treated mice compared to spherical micelle-treated mice at both 8 hrs (~1.8-fold greater, p ≤ 0.001) and 24 hrs (~2.1-fold greater, p ≤ 0.0001).

**Conclusion:**

Overall, this work highlights the promise of a biodegradable anisotropic micelle system to overcome multiple drug delivery challenges and enhance efficacy and safety for the treatment of brain cancer.

## Introduction

Glioblastoma (GBM), a grade IV astrocytoma, is one of the most elusive cancers to current treatment options of radiation and chemotherapy.^1^,^2^ The administration of the commonly used chemotherapeutic drug, Temozolomide (TMZ), in combination with radiation, post resection, has only extended the median lifespan of GBM patients to 15 months with a 5-year survival rate of less than 5% following diagnosis.^3^ A major reason for the high GBM mortality rate is the fast (median 6.9 months) and frequent (90% at original site) recurrence of the tumor.^4^,^5^

It is understood that GBM is highly regulated by the different cell types surrounding the tumor.^6^ Mechanical and chemical signaling promotes recurrence of the tumor and it is difficult to overcome the elaborate pathways and signals involved to attenuate further invasive growth of the tumor.^7^–^12^ After resection, it has been suggested that the tumor cells undergo a shift to a more drug-resistant state, preventing commonly used chemotherapeutic drugs such as temozolomide (TMZ) from having a significant therapeutic effect.^13^,^14^ Therefore, new therapeutics as well as new technologies able to deliver high drug doses to brain tumor cells are needed to provide long-term protection of the microenvironment and prevent tumor recurrence.

Nanotechnology has offered a promising ability to maintain small molecules in blood circulation, reduce clearance through the kidney, and improve biodistribution to a target organ or tissue of interest.^15^ Nanoparticles composed of biodegradable and biocompatible materials can be used to formulate hydrophobic molecules for minimally invasive administration through a single intravenous injection, rather than a prolonged infusion.^16^ Nanoparticles are particularly advantageous for use as medicines because many of the drugs used for treatment of debilitating diseases such as cancer have very low solubility in aqueous solutions, thus requiring a vehicle for efficient delivery and localization to a particular location in the body.^17^ Also, non-spherical nanoparticles with larger aspect ratios, which can be fabricated via either a bottom-up or top-down approach, have been demonstrated to more effectively avoid macrophage uptake and clearance, and have enhanced uptake into cancer cells compared to analogous spherical nanoparticles.^18^,^19^ In addition, the controlled biodegradability of nanoparticles can provide long-term, sustained release of small molecule drugs to tumors to maximize efficacy and decrease required dosing frequency.^20^

A hydrophobic small molecule verteporfin (VP) has recently been shown to attenuate GBM growth and proliferation.^21^ VP is a benzoporphyrin derivative commonly used as a photosensitizer in photodynamic therapy for the treatment of wet-age-related macular degeneration.^22^ Free radicals generated from the treatment are able to suppress blood vessel formation.^23^ Recent studies indicate that VP is also a potent inhibitor of Yes-associated protein (YAP), a key transcriptional coactivator of the Hippo pathway and has been shown to operate as an oncoprotein in cancers.^24^ With YAP being overactive in cancerous tissues, we postulated that VP could show cytotoxic effects to a greater degree on GBM cells in comparison to healthy brain stromal cells,^7^,^10^,^12^,^25^–^27^ as was previously shown for multiple cancers, including pancreatic, small cell lung cancer, triple-negative breast cancer, and brain cancer.^21^,^28^–^31^

In this study, we encapsulated VP in a micelle vehicle composed of poly(ethylene glycol)-poly(β-amino ester)-poly(ethylene glycol) (PEG-PBAE-PEG) triblock copolymer. PEG was used to form the corona of the micelle to provide stealth and avoid non-specific binding to proteins in serum. PBAE is a well-studied cationic, biodegradable polymer with tertiary amines in the backbone that provide excellent buffering capacity for enhancing endosomal escape in the cytosol.^32^–^34^ It was recently shown that PBAE-based micelles can be synthesized into different aspect ratio shapes by altering the PBAE backbone hydrophobicity and PEG molecular weight.^30^ After following a similar protocol and confirming the synthesis of the VP-loaded micelles of two different morphologies, we investigated the effect of VP-encapsulated micelles on two patient-derived GBM cell lines and normal human astrocytes (NHA), representing the non-transformed brain parenchyma. Furthermore, we investigated the effect of micelle morphology on the pharmacokinetics and biodistribution of VP in an ectopic mouse xenograft model.

## Materials and Methods

### Materials

1,4-Butanediol diacrylate (B4), 1,6-hexanediol diacrylate (B6), 1-(3-aminopropyl)-4-methylpiperazine (E7) (Alfa Aesar), octylamine (S8m), decylamine (S10m), acetone, dimethyl sulfoxide (DMSO), dimethylformamide (DMF), tetrahydrofuran (THF), hexane, citric acid monohydrate, disodium phosphate (Sigma-Aldrich, St. Louis, MO, USA), methoxy poly(ethylene glycol) thiol (2 kDa and 800 Da) (Laysan Bio, Inc.), and Verteporfin (VP (US Pharmacopeial Convention, Inc.) were purchased and used as received.

### Cell Culture

All experiments were performed following the relevant guidelines and regulations from the Johns Hopkins University, Mayo Clinic, and the National Institutes of Health. Animal protocols were approved by the Mayo Clinic Animal Care Institutional and Use Committee. GBM patient-derived tumor-initiating cell line, GBM1A, was originally derived and characterized by Vescovi et al.^35^ GBM patient-derived tumor-initiating cell line, JHGBM612, was established from a patient with a butterfly GBM exhibiting pronounced invasive spread, and was characterized by our group previously.^36^ Normal human astrocytes (NHA) were purchased from Lonza (Walkersville, MD, USA). GBM1A and JHGBM612 were cultured as neurospheres in serum-free medium containing DMEM/F-12 (Invitrogen, Carlsbad, CA, USA) and 1% antibiotic/antimycotic, supplemented with B27, 20 ng/mL epidermal growth factor (EGF) and 20 ng/mL basic fibroblast growth factor (bFGF) (Sigma-Aldrich). NHA were cultured in ABM Basal Medium supplemented with AGM SingleQuots Supplements necessary for the growth of astrocytes (Lonza). All cells were grown in an incubator at 37°C and 5% CO_2_.

### Polymer Synthesis

Two amphiphilic triblock copolymers were synthesized via a two-step Michael Addition reaction. 1,4 Butanediol diacrylate (B4) was reacted with octylamine (S8m) by Michael Addition reaction at a molar ratio of 1.15:1 at 90°C for 72 hrs to yield acrylate-terminated hydrophobic (B4S8m) PBAE base polymer. In another reaction, 1,6 hexanediol diacrylate (B6) was reacted with decylamine (S10m) by Michael Addition reaction at a molar ratio of 1.15:1 at 90°C for 24 hrs to yield another acrylate-terminated hydrophobic PBAE base polymer (B6S10m). Both base polymers were precipitated twice in hexane and then dried under vacuum with desiccant overnight. The structure and molecular weight of the base polymers were confirmed using Bruker Avance III 500 MHz^1^H NMR spectrometer in CDCl_3_. Following a protocol described by Kim et al, the B4S8m was then endcapped by thiol-ene Michael Addition with 2 kDa mPEG-thiol in DMSO, using 1-(3-aminopropyl)-4-methyl-piperazine (E7) (1:2.5:0.25 w/w/w) as a primary amine-containing catalyst.^34^ The endcapping reaction was performed for 24 hrs at 50°C while stirring. A rotary evaporator was used to remove DMSO from the product, referred to as PP1. Afterwards, the resulting product was purified through precipitation in hexane, and this was repeated thrice. For B6S10m endcapping, a similar protocol was followed; however, 800 Da mPEG-thiol was used. The structures of PP1 and PP2 were confirmed using ^1^H NMR in CDCl_3_.

### Micelle Synthesis

Spherical VP-loaded micelles (sVPM) and filamentous VP-loaded micelles (fVPM) were synthesized using the following nanoprecipitation protocol. For sVPM synthesis, PP1 was dissolved in DMF at 20 mg/mL and then mixed with an equivalent volume of 1 mg/mL VP solution in DMSO (5 wt% feed ratio). Five hundred microliters of the resulting solution was added slowly dropwise to 1.5 mL of stirring deionized (DI) water at 500 rpm in a scintillation vial. The samples were then immediately sonicated in a water bath sonicator for 1 min before returning to the stir plate for 4 hrs. fVPM were synthesized very similarly; however, PP2 was dissolved in acetone. Unloaded micelles were also prepared by replacing the VP solution in DMSO with pure DMSO. After 4 hrs of spinning, the solution was filtered through a 10-kDa MWCO filter. The filtrate was collected and added to a Sephadex column with Sephadex S-500 High Resolution. The tubes were spun at 800 g for 3 mins. The filtrate was collected and then filtered with a 0.22-μm PTFE syringe filter. Then, the resulting solution was aliquoted into several pre-weighed tubes and lyophilized. A 10% sucrose solution was included with the sVPM as a cryoprotectant.

### Micelle Characterization

Lyophilized sVPM and fVPM were resuspended at 1 mg/mL in DI water and then sized using dynamic light scattering (DLS) with Malvern Zetasizer Nano ZS (Malvern Instruments, Malvern, U.K.). In addition, Zetasizer was used to measure the zeta potential of the micelles in 10 mM NaCl at 1 mg/mL. Moreover, transmission electron microscopy (TEM) was used to confirm size and examine the morphology of the micelles. Micelles were resuspended in DI water at 1 mg/mL and then pipetted gently onto a carbon grid (10 μL). The grid was left until the solution fully evaporated and then was submerged for in 0.5% uranyl acetate for 30 sec, then in DI water, and left to fully dry. The grids were then imaged using a Philips/FEI BioTwin CM120 TEM.

### VPM Loading Capacity

VP was released from micelle core by dissolving lyophilized micelles in DMSO at 1 mg/mL. The resulting solution was then added to a dark 96-well plate, and several serial half dilutions were made. The fluorescence signals of the wells were measured with a Synergy 2 plate reader (BioTek) at an excitation wavelength of 420 nm and an emission wavelength of 680 nm (n=3). VP concentration was then determined using a standard curve to interpret the fluorescence intensity values. The VP loading capacity (LC) and loading efficiency (LE) were then calculated according to the following formulas:

$${\rm LC{(\%)}={{{mass}\;{of}\;{VP}}\over{{mass}\;{of}\;{polymer}}} x100}$$

$${\rm {LE}{(\%)}={{{yielded}\;{VP}\;{mass}}\over{{fed}\;{VP}\;{mass}}} x100$$

### Evaluation of Solubility

To measure VP solubility in both the unencapsulated and micelle-encapsulated forms, samples were mixed with 1× PBS at an equivalent concentration of 2 mg/mL VP. Equivalent volumes (50 μL) were added into a 96-well plate, and turbidity (absorbance) was measured with a plate reader at 550 nm (n=3).

### VP Release Kinetics

Micelles were resuspended at 1 mg/mL inside citrate-phosphate buffers maintaining pH at 5, 6.5, and 7.4 to simulate the local environments of endosomes, extracellular tumor tissue, and normal blood vessels, respectively. Buffers were produced by mixing 0.1 M citric acid monohydrate and 0.2 M disodium phosphate at different v/v ratios to produce the desired pHs. One milliliter of resuspended micelles was kept at 37°C in between timepoints (n=3). At 1, 3, 5, and 10 hrs timepoints, micelle solutions were centrifuged at 200,000 ﻿rpm for 20 mins at 4°C. Eight hundred microliters of the supernatant was removed and replaced with a fresh buffer of the same pH. The micelle pellet was resuspended and returned to 37°C until future timepoints. The collected supernatants were frozen, lyophilized and then resuspended with DMSO to solubilize the VP in the release samples. The samples were centrifuged at 15,000 rpm to pellet the buffer salts, but leave the solubilized VP in the supernatant. After centrifuging, the supernatant of each sample was added to a dark, flat-bottom 96-well plate and then fluorescence intensity was measured with a plate reader (420 nm excitation, 680 nm emission wavelengths). The concentration of VP in each tube was determined using a pH-specific standard curve previously developed.

### Cellular Uptake

GBM1A, JHGBM612, and NHA cells were seeded at 15,000 cells per well in 96-well plates in 100 μL of media, and incubated in an atmosphere of 5% CO_2_ at 37°C for 24 hrs. Equivalent final VP concentrations of free VP, fVPM, and sVPM ranging from 7.8125 to 125.0 nM were incubated with the cells for 1.5 hrs (n=4). After incubation, cells were then washed three times with heparin in 1× PBS (50 μg/mL) to remove VP possibly bound electrostatically to the cell membranes, trypsinized, neutralized with FACS buffer (2% FBS in 1× PBS), transferred to a round-bottom 96-well plate, centrifuged, resuspended with FACS buffer, and then analyzed by flow cytometry (BD Accuri C6 with HyperCyt adaptor). The results were then analyzed by FlowJo 7.6.5 software using FSC-H vs SSC-H gating for singlet cells and FL3 vs FSC-H gating for VP-positive cells. All wells included in data analysis had at least 500 singlet events per well.

### Cell Viability

GBM1A, JHGBM612, and NHA cells were seeded at 15,000 cells per well in 96 well plates in 100 μL of media, and incubated in an atmosphere of 5% CO_2_ at 37°C for 24 hrs. Equivalent final VP concentrations of free VP, fVPM, and sVPM ranging from 3.90625 to 500.0 nM were incubated with the cells for 2 hrs (n=4). Following treatment, cells were washed 3 times with 1× PBS, and incubated for 24 hrs in 100 μL of fresh media. Viability was then determined by incubating the cells with 100 μL of CellTiter 96AqueousOne MTS assay solution in complete media (1:5 v/v) in an atmosphere of 5% CO_2_ at 37°C for 2 hrs. The resulting absorbance of the wells was measured using a plate reader at 490 nm. Brightfield images of each condition were taken right before incubation with MTS assay solution.

### VPM Pharmacokinetics and Biodistribution

All animal protocols were approved by the Mayo Clinic Animal Care Institutional and Use Committee. Athymic nude mice 5–7 weeks of age were inoculated with 2 × 10^6^ human glioblastoma (GBM1A) cells in matrigel solution in the flank. Once tumors reached 150 mm^3^, they were randomized into four different groups (n=4 each) for blood half-life and tissue distribution studies. For each study, one group was injected in tail-vein with spherical micelles while the other group was injected with filamentous micelles. These micelles were formulated following the protocol above with near-infrared (NIR)-dye (Lumiprobe) encapsulated in place of VP for fluorescence imaging, lyophilized, and reconstituted to 1.75 mg/mL of NIR-dye prior to injection. To study pharmacokinetics, blood was collected from the saphenous vein at 5, 10, 30 mins, 1, 2, 4, and 8 hrs timepoints post-injection into heparinized capillary tubes. Fluorescence in capillary tubes was imaged using IVIS. To study biodistribution, the whole animal live image was acquired using IVIS at 0, 0.5, 1, 2, 4, 8, and 24 hrs post-injection. Then, animals were sacrificed at 24 hrs and organs (liver, spleen, kidneys, bladder, lungs, and heart) were harvested for imaging individually with IVIS. An ROI was drawn around each organ and fluorescence intensity was quantified with Living Image 3.2 software. To calculate percent distribution, each fluorescence intensity value was normalized to the sum of fluorescence intensity values from all measured organs.

### Statistics

GraphPad Prism 6 software package was used to perform statistical analysis. One-way ANOVA with Bonferroni post hoc test was used to compare all pairs, or Student’s *t*-test was used to compare two conditions. Two-way ANOVA with Tukey post hoc test was used for multiple comparisons (*p ≤ 0.05, **p ≤ 0.01, ***p ≤ 0.001, ****p ≤ 0.0001). p-values less than 0.05 were considered statistically significant.

## Results and Discussion

### Synthesis and Characterization of PBAE Base Polymer and Triblock PEG-PBAE-PEG Copolymer

PBAE base polymers were synthesized via Michael Addition (1.15:1 ratio of diacrylate to alkylamine monomer). Resulting molecular weights for B4S8m and B6S10m were 5274 Da and 6471 Da, respectively. As reported in related studies of particle thermodynamics and assembly, the greater hydrophobicity of B6S10m compared to B4S8m increases the critical packing parameter (*v*/*α*_0_*l*_c_, where *v* is the volume of the hydrocarbon core, *α*_0_ is the effective head group area, and *l*_c_ is the hydrocarbon chain length), producing more wormlike micelles.^37^ B4S8m and B6S10m were end-capped with 2000 and 800 Da mPEG-thiol via Michael Addition to synthesize PEGylated polymers, PP1 and PP2, respectively. By endcapping B6S10m with a lower molecular weight PEG compared to B4S8m, this decreased the critical packing parameter variable, *α*_0_, to increase the overall critical packing parameter and generate a more filamentous shape. The reactions were confirmed through 1H NMR spectrum in Figures S1A and B. The PP1 and PP2 spectrum show reduced area for peaks a–c, which correspond to hydrogens of the base polymer diacrylates. The reaction efficiencies were 77% and 67% for PP1 and PP2, respectively.

### Micelle Characterization

**Figure 1:**
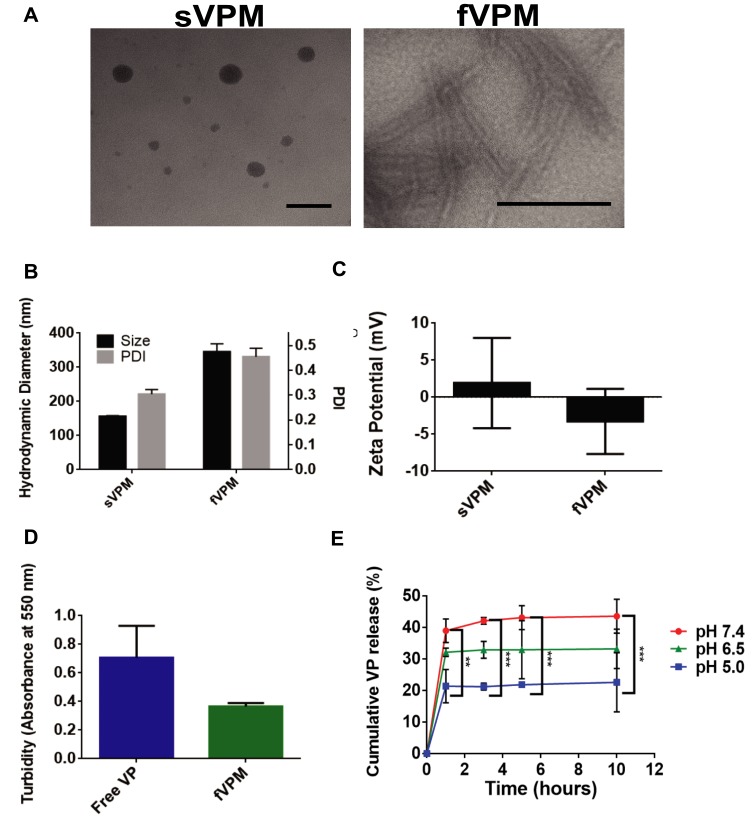
Triblock copolymer micelles loaded with VP exhibit tunable morphology, pH-sensitivity, and aqueous solubility. (**A**) Representative TEM images of both sVPM (left, scale bar= 100 nm) and fVPM (right, scale bar= 500 nm), (**B**) size and polydispersity index (PDI) of micelles (n=3, mean ± SD), (**C**) zeta potential of sVPM and fVPM in 10 mM NaCl, (**D**) turbidity measured with both free and encapsulated VP in fVPM at 2 mg/mL VP in 1× PBS (n=3, mean ± SD), (**E**) VP release from fVPM at 0, 1, 3, 5, and 10 hrs timepoints in buffers prepared at pH 5.0, 6.5, and 7.4 (n=3, mean ± SD, one-way ANOVA with Bonferroni’s multiple comparisons test (**p ≤ 0.01, ***p ≤ 0.001)).

Micelles were synthesized via nanoprecipitation and subsequently characterized in terms of size, shape and surface charge. TEM images in Figure 1A show that sVPM were spherical, while fVPM had a filamentous, wormlike shape. These morphologies and aspect ratios were highly reproducible and did not exhibit batch-to-batch variability. As shown in Figure 1B, DLS determined that the sphere-equivalent hydrodynamic diameters for sVPM and fVPM were 156 ± 2 nm and 350 ± 20 nm, respectively. Given that these are intensity-weighted hydrodynamic diameters, TEM exhibited particles of a smaller size, particularly for sVPM. In addition, the PDI values were 0.30 ± 0.02 and 0.46 ± 0.03, respectively. The larger aspect ratio for fVPM can explain the larger size and PDI compared to sVPM, given that DLS calculates size assuming spherical particles in the Stokes–Einstein equation. The zeta potentials (Figure 1C) for sVPM and fVPM in 10 mM NaCl were 2 ± 6 mV and −3 ± 4 mV, corroborating that PEG is on the surface of both micelles due to the neutral charge measured. The LC was determined to be 5.62% and 6.82%, for sVPM and fVPM, respectively. The LC differed less than 1% between repeated batches of both formulations. Although the initial feed ratio of VP to polymer was 5 wt%, a >5% LC most likely occurs due to a loss of polymer in the post-synthesis centrifuging and filtering steps. The LE was determined to be 33.7% and 14.6%, for sVPM and fVPM, respectively. The lower polymer yield for fVPM (~10%) compared to sVPM (~25%) can explain the greater LC, but lower LE. It is possible that the longer PBAE backbone for fVPM may prevent some hydrophobic chains from organizing in the core of the micelle, leading to more unstable self-assemblies.

### Solubility Enhancement

To overcome a very poor water solubility, hydrophobic VP was encapsulated in micelles with a hydrophobic interior and hydrophilic PEG exterior. Solubility enhancement was determined by analyzing the turbidity of PBS solutions with free VP or an equivalent mass of VP encapsulated inside micelles. Absorbance/turbidity was measured at a peak wavelength of VP absorption/turbidity (550 nm). Figure 1D shows lower absorbance for encapsulated VP compared to free VP. The very low solubility of free VP in PBS results in high turbidity which decreases light penetration, increasing the measurement value for absorbance. The greater solubility of micelles in PBS creates a clearer, more homogeneous solution, allowing more light to penetrate through the sample, decreasing the level of absorbance/turbidity measurement. This confirms that VP-encapsulation inside micelles that display hydrophilic PEG chains on the corona enhances the solubility of VP, and provides a more usable and practical formulation for an in vivo environment.

### pH-Sensitive Release of VP from fVPM

PBAE polymers have tertiary amines in their backbones which provide excellent buffering capacity in an acidic environment, leading to efficient endosomal escape inside cells. pH-sensitive release was evaluated by resuspending fVPM at 1 mg/mL inside citrate-phosphate buffers at pH 5, 6.5, and 7.4 in order to simulate the local environments of endosomes, extracellular tumor microenvironment, and blood vessels, respectively. Figure 1E shows the release plots at all three pH values over 10 hrs. All three plots show similar kinetic trends, but differing percentages of total VP mass released with pH 7.4 showing the greatest mass of VP release, pH 6.5 showing an intermediate VP mass release, and pH 5.0 showing the lowest percentage of VP released. This trend is comparable with that observed by Tzeng and Green, in which PBAE polyplexes were used.^38^ Table 1 summarizes the key chemical and physical characteristics of sVPM and fVPM.

**Table 1:** Summarized Chemical and Physical Characteristics of sVPM and fVPM

Chemical/Physical Characteristics	sVPM	fVPM
PBAE base polymer/MW (Da)	B4S8m/5274	B6S10m/6471
PEG-SH MW (Da)	2000	800
Morphology	Spherical	Filamentous
Hydrodynamic diameter (nm)	156 ± 2	350 ± 20
PDI	0.30 ± 0.02	0.46 ± 0.03
Zeta potential (mV)	2 ± 6	−3 ± 4
Loading capacity (%)	5.62	6.82
Loading efficiency (%)	33.7	14.6

### VP Internalization in Patient-Derived Glioma and Astrocyte Cells

**Figure 2:**
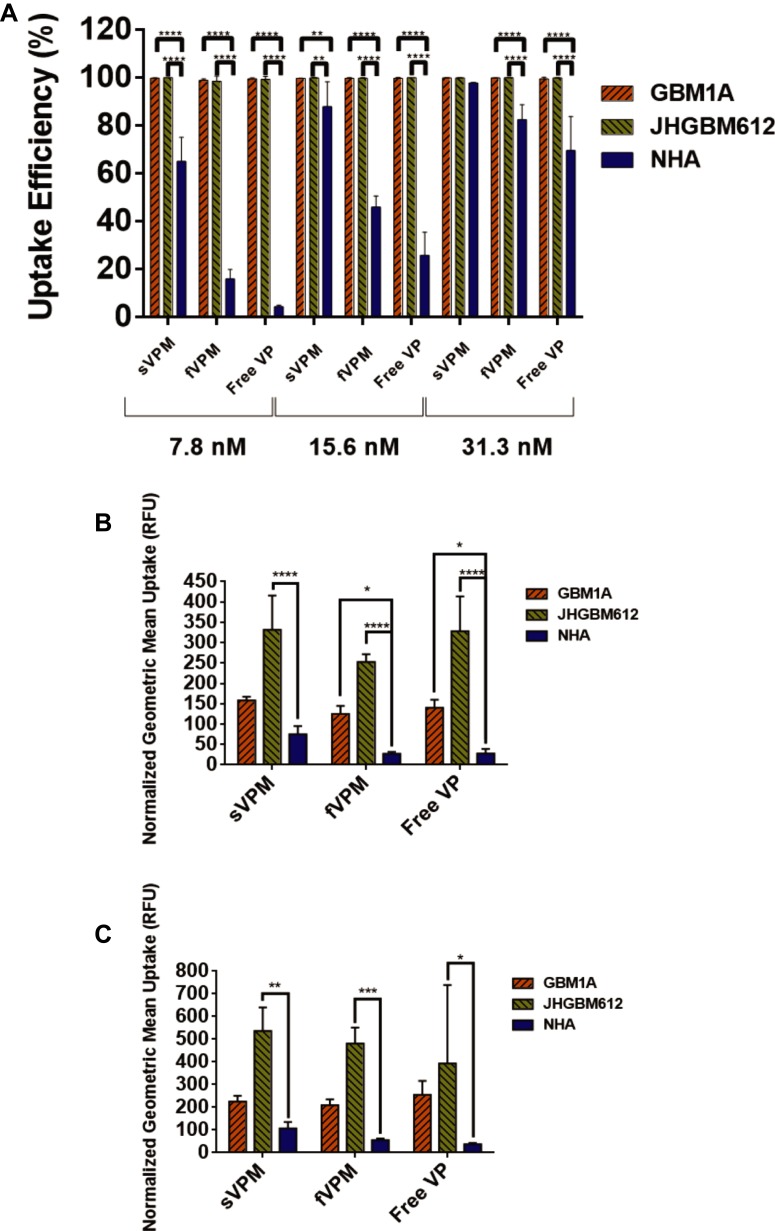
NHA demonstrate significantly lower uptake efficiency and normalized geometric mean uptake of free VP, sVPM, and fVPM compared to patient-derived GBMs. (**A**) Consolidated uptake efficiency data from all three cell types and treatments at 7.8, 15.6, and 31.3 nM (n=4, mean ± SD, one-way ANOVA with Bonferroni’s multiple comparisons test (**p ≤ 0.01, ****p≤ 0.0001)). (**B** and **C**) Consolidated normalized geometric mean measurements at 62.5 nM (**B**) and 125 nM (**C**) VP treatment for all three tested cell types (n=4, mean ± SD, one-way ANOVA with Bonferroni’s multiple comparisons test (*p ≤ 0.05, **p ≤ 0.01, ***p ≤ 0.001, ****p ≤ 0.0001)). Cells were normalized to untreated controls of the same cell type.

To evaluate the level of VP-loaded micelle and free VP uptake in GBM1A, JHGBM612, and NHA, flow cytometry was performed given the fluorescence property of VP. Cells were incubated with VP at equivalent concentrations ranging from 7.81 to 125 nM for 1.5 hrs. Uptake efficiency was first measured to determine the number of cells out of the whole population that showed positive fluorescence in the PE channel. We did not observe cell death due to careful preparation of the samples. In addition, no cytotoxicity was expected from the VP treatment given the very short incubation time. As shown in Figure S2A and C, nearly 100% of cells had successful uptake for all formulations, sVPM, fVPM, and free VP, at every concentration in GBM1A and JHGBM612. In Figure S2E, NHA exhibits a dose-dependent uptake efficiency, with sVPM showing the highest uptake efficiency, followed by fVPM and then free VP. Cellular uptake saturation was observed for all treatments at approximately 62.5 nM. Geometric mean of fluorescence intensity was also evaluated to measure the relative amounts of VP entering each cell. The intensity was analyzed only from the VP+ cells in order to eliminate bias from uptake efficiency. The results shown in Figure S2B, D and F demonstrate that VP per cell increased in a dose-dependent manner for all cell types and for all treatments. Figure S2G and H highlight the statistically significant differences in the averaged geometric mean uptake for all three formulations between different cell types for 62.5 and 125 nM treatments, respectively. Figure 2A demonstrates significantly lower uptake efficiencies in NHA compared to GBM1A and JHGBM612 for all formulations treated from 7.81 to 31.3 nM, except sVPM at 31.3 nM. Figure 2B and C show that the normalized uptake values for NHA are very low compared to GBM1A and JHGBM612. For example, statistical analysis of fVPM uptake at 62.5 nM VP showed that the differences between GBM1A and NHA (p ≤ 0.05) and JHGBM612 and NHA (p ≤ 0.0001) were significant. At 125 nM, fVPM uptake was significantly higher only in JHGBM612 in comparison to NHA (p ≤ 0.001). For all cell types and the majority of tested concentrations, sVPM showed the greatest geometric mean, suggesting a larger VP dosage entering the cells compared to fVPM or free VP. It has been demonstrated that cancer cell uptake of spherical-shaped nanoparticles happens more readily than larger aspect ratio nanoparticles.^18^,^39^–^41^ It is postulated that rod-shaped nanoparticles internalize slower into cells because their contact angle in relation to the cell membrane can vary, whereas spherical particles, which are symmetrical, have a constant contact angle to the cell membrane. For example, if a rod-shaped particle approaches a cell with its long axis parallel to the cell membrane, it is more difficult for the cell to “wrap” around the particle.^42^ Moreover, the high uptake efficiencies suggest that the PEG corona on these micelles may still permit some level of protein adsorption onto the micelle surface. This can enhance electrostatic interaction between the micelles and cell membranes, increasing endocytosis of the micelles. It has been reported previously that grafting 3.4-kDa PEG chains onto polystyrene nanoparticles demonstrated 90% reduction of protein adsorption compared to uncoated nanoparticles.^43^ Thus, the lower molecular weight PEG used in these studies would most likely permit a greater level of protein adsorption. In addition, macropinocytosis may play a role in this uptake.

### VP-Induced Cell Death in Patient-Derived Glioma and Astrocyte Cells

**Figure 3:**
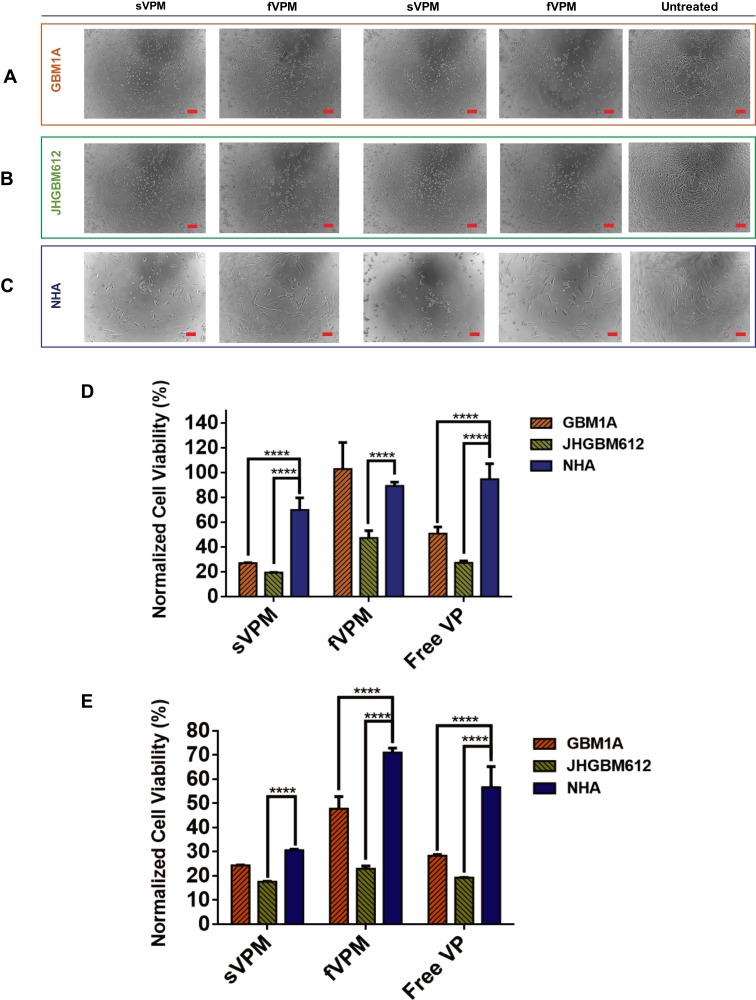
NHA viability is significantly greater than patient-derived GBMs after free VP, sVPM, and fVPM treatments. (**A**–**C**) Representative brightfield images for corresponding cell types after 62.5 nM VP (left), 125 nM VP (center) and no treatment (right) (scale bar = 100 µm). (**D** and **E**) Consolidated normalized cell viability measurements at 62.5 nM (**D**) and 125 nM (**E**) VP treatment for all three tested cell types (n=4, mean ± SD, one-way ANOVA with Bonferroni’s multiple comparisons test (****p ≤ 0.0001)). Cells were normalized to untreated controls of the same cell type.

In order to evaluate the potency of VP as a chemotherapeutic for patient-derived GBM cell lines and its effect on NHA, sVPM, fVPM, and free VP were added to GBM1A, JHGBM612, and NHA for a 2 hr treatment period at final VP concentrations between 3.9 and 500 nM. And, 24 hrs later, the cells were imaged (Figure 3A–C) and their metabolic activities were measured with MTS. At 15.6 nM and below, there was no cytotoxicity observed for any of the cell types. Interestingly, 62.5 nM and/or 125 nM VP treatments displayed the greatest cytotoxicity differential among the three different treatments in all three cell types. Figure 3D shows that at 62.5 nM, sVPM-treated GBM1A, JHGBM612, and NHA exhibited viabilities of 27%, 19%, and 70%, respectively. For fVPM treatment, viability was 103%, 47%, and 89%, respectively. And for free VP, viability was 51%, 27%, and 95%, respectively. The difference in viability was significant between individual GBM lines and NHA (p ≤ 0.0001), for all conditions and treatments, except fVPM-treated GBM1A cells and NHA. fVPM did not show greater cytotoxicity in any of the cell lines compared to sVPM, at both 62.5 and 125 nM. Figure 3E shows that at 125 nM, sVPM-treated GBM1A, JHGBM612, and NHA exhibited viability levels of 24%, 18%, and 31%, respectively. At 125 nM fVPM treatment, viability levels were 48%, 23%, and 71% in GBM1A, JHGBM612, and NHA, respectively. For 125 nM free VP, similar viability values were observed for GBM1A (28%) and JHGBM612 (19%); however, for NHA, viability increased to 57%. For 125 nM, all comparisons between GBM1A and NHA, and JHGBM612 and NHA were statistically significant (p ≤ 0.0001) for sVPM, fVPM, and free VP, except for sVPM between GBM1A and NHA. The shown brightfield images in Figure 3A–C align with the viability values aforementioned. Moreover, Figure S3A–C displays the comprehensive viabilities for all three cell types after sVPM, fVPM, or free VP treatments. Regarding potential clinical translation, while fVPM does not achieve as high killing of GBM cells as is observed for sVPM, the combination of diminished cytotoxicity towards NHA and the advantageous pharmacokinetic and biodistribution properties of these micelles may provide several therapeutic benefits over sVPM for future in vivo treatments.

In addition, as shown in Figure S3D and E, the difference in average normalized cell viability for all three treatments between the three different cell types at 62.5 nM and 125 nM VP is statistically significant. For 62.5 nM VP treatment, there is an average 24% higher cell viability for NHA compared to GBM1A (p ≤ 0.0001), and 53% higher cell viability for NHA compared to JHGBM612 (p ≤ 0.0001). The differing cytotoxicity observed in the treatments between GBM cells and NHA can be rationalized by the varying levels of VP entering each cell type. According to the normalized geometric mean fluorescence intensity for cellular uptake results in Figure S2G (62.5 nM) and H (125 nM), the difference among all three treatments is statistically significant when comparing both GBM1A and NHA (p ≤ 0.001, 0.05), and JHGBM612 and NHA (p ≤ 0.0001 for both). More specifically, GBM1A cells have roughly more than two-fold greater VP uptake per cell for all formulations compared with NHA, and JHGBM612 cells have approximately six-fold greater VP uptake per cell compared to NHA.

Previous studies have demonstrated that relative amounts of nanoparticle uptake between different cell types is dependent on the cell size and shape.^44^,^45^ Astrocytes exhibit longer neurite-like projections and a more stretched appearance compared to the patient-derived GBM cell lines used in these studies. It is possible that the astrocyte neurites might sterically hinder nanoparticle uptake into the cell body and cause other differences in endocytosis. Furthermore, it is known that the highly invasive nature of GBM cells is aided by the degradation of the cancer cell extracellular matrix (ECM) via protease secretions.^46^ Reduced ECM surrounding GBM cells compared to astrocytes may enhance the uptake of the micelles into GBM cells. In addition to enhanced uptake into the cancer cells, the YAP-TEAD pathway, the primary target of VP, is activated more in cancer cells compared to healthy cells. This may enhance the ability for VP to elicit a heightened cytotoxic effect in GBM cells compared to NHA. It is important to note that the NHA cell line, which is established from a non-neoplastic source, may not necessarily represent similar molecular pathway activation such as YAP-TEAD4 signaling, observed in tumor-associated astrocytes. These tumor-surrounding glial cells have been shown to undergo phenotypic and genotypic alterations compared to those in a normal, healthy brain parenchyma.^47^ While further investigation is warranted to identify the mechanisms involved, it is important to highlight that the more cytotoxic nature of all VP treatments against GBM cells compared to astrocytes provides a critical therapeutic window to kill the cancer cells while attenuating the same damage against the healthy brain parenchyma.

### Pharmacokinetics and Biodistribution in Ectopic Mouse Xenograft Tumor Model

**Figure 4:**
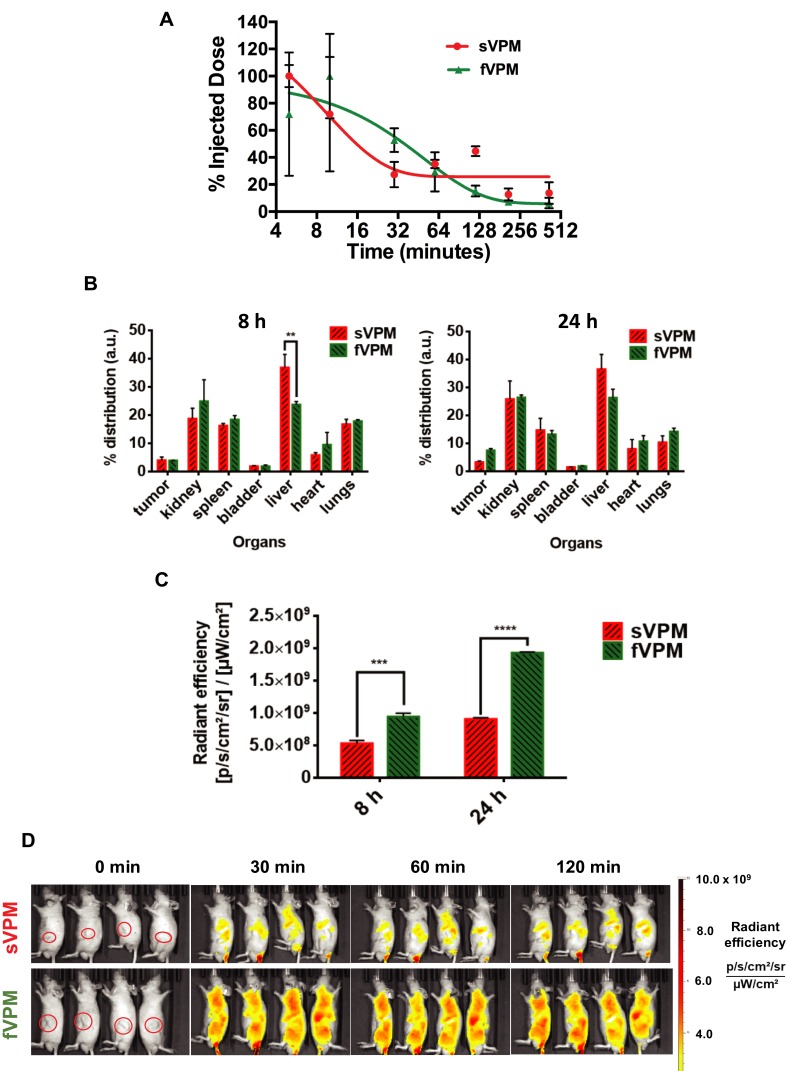
fVPM exhibit enhanced pharmacokinetic properties compared to sVPM in an ectopic human GBM tumor mouse flank model. (**A**) sVPM and fVPM in blood at 5, 10, 30, 60, 120, 210, and 420 min timepoints, (**B**) 8 hrs and 24 hrs biodistribution for tumors and major organs (n=2, mean ± SD, one-way ANOVA with Bonferroni’s multiple comparisons test (**p ≤ 0.01)). (**C**) Nanoparticle signal in tumors after 8 and 24 hrs (n=2, mean ± SD, one-way ANOVA with Bonferroni’s multiple comparisons test (***p ≤ 0.001, ****p ≤ 0.0001)). (**D**) sVPM and fVPM- treated animals under IVIS after 0 min, 30 mins, 60 mins, and 120 mins. Animals in the same image are replicates. Red circles in leftmost panels indicate the location of tumors.

Our group has previously demonstrated that filamentous micelles are able to avoid macrophage uptake more effectively than spherical micelles in vitro.^30^ To test the capability of fVPM to evade macrophages and provide prolonged systemic circulation in vivo in comparison to its spherical counterpart, both fVPM and sVPM were systemically injected in mice with GBM tumors in the flank. Blood samples were taken over the course of 7 hrs to evaluate the clearance of micelles. Figure 4A shows that fVPM were cleared more slowly than sVPM. fVPM had a half-life of approximately 35 mins, whereas the half-life of sVPM was calculated to be approximately 6.6 mins. A longer blood circulation will increase the likelihood of the micelles to enter the tumor microenvironment via leaky vasculature following repeated flow-through in systemic circulation.

To determine the localization of sVPM and fVPM to the tumor, NIR dye-loaded micelles were injected via the tail vein. The animals were sacrificed after 8 and 24 hrs, and their organs were harvested and read under IVIS to measure emitted signal. Figure 4B shows that the percent biodistribution in the tumors after 8 hrs was 3.92% and 3.91% for sVPM and fVPM, respectively. After 24 hrs, the biodistribution in the tumors was 3.26% and 7.43% for sVPM and fVPM, respectively. Although not a statistically significant difference, the more than double tumor accumulation for fVPM is promising. While comparable tumor accumulation was observed between both micelles after 8 hrs, there was significantly more accumulation in the liver from sVPM compared to fVPM (p ≤ 0.01), suggesting that there may be a lower clearance likelihood of the fVPM compared to sVPM in circulation. Signal from the micelles in the tumors (Figure 4C) shows that there was a statistically significant difference in absolute tumor signal between mice treated with sVPM and fVPM. After 8 hrs, the radiant efficiencies in the tumors were 5.28 × 10^8^ and 9.43 × 10^8^ (p/s/cm^2^/sr)/(µW/cm^2^) for sVPM and fVPM-treated mice (~1.8-fold difference), respectively (p ≤ 0.001). After 24 hrs, the radiant efficiencies in the tumors were 9.06 × 10^8^ and 1.93 × 10^9^ (p/s/cm^2^/sr)/(µW/cm^2^), for sVPM and fVPM-treated mice (~2.1-fold difference), respectively (p ≤ 0.0001). As depicted in Figure S4A and B, significant differences in micelle accumulation normalized to organ mass were measured only in the spleen at 24 hrs.

Based on these in vivo results, the engineered filamentous micelle morphology was observed to provide longer circulation time and partial avoidance of critical clearance mechanisms. Figure 4D highlights this sharp difference with IVIS images of the treated animals. To further improve tumor accumulation, an active targeting moiety, such as hyaluronic acid or folic acid, could be incorporated into the micelle design to target receptors overexpressed on GBM cells (ie CD44 and folate, respectively).^48^,^49^ These ligands can be conjugated to the micelle surface in efforts to improve the chemotherapeutic impact and translational potential of these micelles. Furthermore, while the ectopic subcutaneous tumor model used in this work does not fully recapitulate all of the characteristics of an orthotopic model such as the surrounding brain parenchyma and blood–brain barrier (BBB), it does allow for investigation and analysis of the pharmacokinetics and biodistribution of differently shaped micelles in an in vivo tumor model.

## Conclusion

This study investigated engineered verteporfin (VP)-loaded spherical and filamentous micelles for use as a safe therapy for GBM. By following thermodynamic principles of self-assembly, we engineered triblock copolymers accordingly to generate specifically shaped structures.^37^ As anticipated, a more hydrophobic backbone in combination with a smaller PEG molecular weight yielded a high aspect ratio, wormlike morphology. For spherical and filamentous micelles encapsulating VP, significantly lower VP uptake and induced cytotoxicity was found in healthy human astrocytes (NHA) compared to GBM cells. In addition, an ectopic mouse xenograft tumor model was utilized for comparing pharmacokinetics and biodistribution of spherical and filamentous micelles. Filamentous micelles had a half-life of approximately 35 mins in comparison to the 6.6 min half-life of the spherical micelles. fVPM tumor accumulation was significantly greater at both 8 hrs (~1.8-fold greater) and 24 hrs (~2.1-fold greater) compared to sVPM. The filamentous micelle system demonstrates an ability to circumvent systemic barriers that limit nanoparticle-mediated delivery. Collectively, this work demonstrates the beneficial therapeutic properties of VP-based filamentous micelles for the management and treatment of GBMs with the potential to extend patient survival and quality of life.
